# Can the Internet of Things Persuade Me? An Investigation Into Power Dynamics in Human-Internet of Things Interaction

**DOI:** 10.3389/fpsyg.2022.883110

**Published:** 2022-06-28

**Authors:** Hyunjin Kang, Ki Joon Kim, Sai Wang

**Affiliations:** ^1^Wee Kim Wee School of Communication and Information, Nanyang Technological University, Singapore, Singapore; ^2^Department of Media and Communication, City University of Hong Kong, Kowloon, Hong Kong SAR, China; ^3^Department of Communication Studies, Hong Kong Baptist University, Kowloon, Hong Kong SAR, China

**Keywords:** internet of things, persuasion, reactance, social power, smart object, mobile application

## Abstract

The advent of artificial intelligence (AI) and the Internet of Things (IoT) has revolutionized user experience with objects. *Things* can perform social roles and convey persuasive messages to users, posing an important research question for communication and human-computer interaction researchers: *What are the factors and underlying mechanisms that shape persuasive effects of IoT?* Bridging the reactance theory and the computers are social actors paradigm, this study focuses on how power dynamics are shaped in human-IoT interactions and its implications on persuasion. Specifically, the study examines the effects of the social role assigned to the IoT mobile app agent and the scope of IoT controlled by the app on users’ perceived power and subsequent persuasive outcomes. The results reveal that when the mobile IoT app is for controlling a smart home, the servant (vs. companion) agent elicits greater perceived power over IoT for users, leading to less threat-to-freedom and better persuasive outcomes, including attitude, intention, and actual behavior. However, such a difference is not observed when the mobile app is for controlling a single smart device (i.e., smart fridge). The study findings offer valuable implications for communication practitioners interested in using IoT as a persuasive tool.

## Introduction

Internet of Things (IoT) has reshaped user experience with everyday objects (Hoffman and Novak, 2018). Users can create their own IoT ecosystem that fits individual needs and lifestyles by connecting a wide range of technological components, including smart objects, cloud networks, and communication devices. Everyday objects have become increasingly smart with their artificial intelligence (AI) capabilities, autonomously collecting and analyzing data from users and surrounding environments to achieve the goals.

In general, one of the communication devices connected to the IoT system becomes the central hub that links users with the system. This hub technology helps users conveniently monitor and control the connected devices, thereby becoming the most proximate source that directly communicates with its user. Mobile applications, often integrating AI agents such as Siri or Bixby, are common tools that function as a connecting nod in human-IoT interactions. This context-aware technology can provide highly personalized messages—from simple notifications to promotional messages—that may influence user behaviors. Hence, in the perspectives of users, they are no longer perceived merely as inanimate *passive things*, but as *active beings* capable of exercising their agency. This also suggests that smart objects can function as autonomous sources, not just conduits, of information in the traditional SMCR (Sender-Message-Channel-Receiver) chain of communication (Berlo, 1960). In other words, user-product (object) communications have become possible in the IoT environment. This revolutionary change in user behaviors that IoT has brought poses a series of interesting research questions for both communication researchers and practitioners: *Can a message from IoT influence how users think and behave?* If so, *what are the factors and underlying mechanisms of the effects?*

To answer these questions, the current study focuses on the sense of power that users may experience in interactions with smart things. In social relationships, power refers to one’s capacity to influence another (French and Raven, 1959). As IoT becomes capable of exercising its agency along with anthropomorphic cues attached to them (e.g., voice, face, and conversational language), users tend to treat them as social actors (Reeves and Nass, 1996) and build human-like relationships that involve power dynamics. In addition, users are likely to experience different levels of power in the interactions with IoT, depending on the kind of social roles played by the IoT agent and the degree of power users have over IoT. As such, this study investigates whether different social roles (i.e., servant vs. companion) assigned to the IoT mobile app agent affect users’ perceived social power and how the scope of IoT that the agent governs (i.e., smart home vs. smart object) moderates this process. By incorporating the reactance theory (Brehm, 1966), we further explore how the social power that users experience with the IoT agent shapes perceptions of threat-to-freedom and the subsequent persuasive impact of the messages delivered by that agent. The findings of this study will offer useful implications for communication practitioners interested in the use and effect of pervasive technology (e.g., IoT) as a persuasive tool.

## Literature Review

### Power Relationships Between Users and Internet of Things

The advent of IoT technology has facilitated everyday objects and devices, such as smart refrigerators, home voice controllers, and smartwatches, to satisfy users’ needs and preferences. These intelligent objects are capable of collecting and analyzing data from users and their environments, which enable them to exercise their agency by acting on specific goals autonomously and making decisions accordingly (e.g., providing personalized recommendations), thereby imbuing a sense of agency among users (Sundar and Nass, 2001; Kim, 2016).

In addition, a smart object is often embodied as an agent (e.g., Siri, Bixby) with human-like characteristics (e.g., facial features, voice, conversational tone). The anthropomorphic cues embedded in smart objects reinforce the socialness of human-IoT interactions (Sundar et al., 2015). For example, Jia et al. (2013) found that people tend to perceive a speaking tissue box (e.g., saying “Bless You” to a sneezing person) as agentic and social as a human uttering the same words. Kang and Kim (2020) also showed that the inclusion of anthropomorphic features in a smart object exercising its own agency increased a sense of connectedness to the object and, consequently, elicited more positive user responses. These findings suggest that the perception of the agency is likely to be heightened when anthropomorphic cues are attached to smart objects.

According to the computers are social actors (CASA) paradigm, people tend to apply social rules of human-human interaction to interactions with computers (e.g., IoT) (Reeves and Nass, 1996). The tendency to mindlessly follow social rules and expectations when interacting with technologies may lead users to perceive the anthropomorphized smart object as an authentic social actor that can influence themselves. By extension, in the context of human-IoT interactions, IoT-enabled objects with cues signaling human-like characteristics can be perceived as a social actor with varying social power.

French and Raven (1959, p. 150) define power as one’s potential ability to influence a target by eliciting changes in “behavior, opinions, attitudes, goals, needs, values, and all other aspects of the person’s psychological field.” Studies have suggested that social power deriving from different social heuristics and motivations (e.g., reward, coercion, legitimate; French and Raven, 1959) influences one’s decision-making process in various interactions (Keltner et al., 2008).

Internet of things with agentic capacity can provide proxy agency to users by allowing them to precisely control how it works, thus enhancing users’ capacity to influence IoT. However, IoT agency also results in the essential tension between human and machine agency (Sundar, 2020). Prior studies showed that people might experience psychological tension when interacting with inanimate objects exercising their own agency (e.g., Jia et al., 2012, 2013; Sundar, 2020). Given that humans generally desire to control their environment with their own free will (White, 1959), interacting with inanimate agents who exercise own agency may make people feel that their freedom is under threat. Considering the agency tension in human-IoT interactions, we posit that perceived power in users vs. power in IoT would be reversely related; when power in IoT increases, power in users will be compromised, resulting in human-IoT power dynamics. This study thus attempts to understand how social power is formulated when users interact with IoT and its implications on persuasive effects.

### Internet of Things Having Different Social Roles

The social role of machines is an important anthropomorphic feature that plays an integral role in shaping user perceptions of the machine and the information conveyed from it through predefining the power relationships between user and machine. Social role refers to “a socially defined pattern of behavior enacted by a person in a particular social position or belonging to a particular social category” (Bosak, 2018, p. 3). As such, the dynamics of social life are shaped by the social roles played by social actors. When it comes to human-IoT interaction, some evidence shows that the voice intelligence system with female voices, such as Alexa and Siri, intensifies existing gender stereotypes—female as an assistant or female having less power (West et al., 2019). Such perceptions of social roles attached to digital devices suggest that people indeed perceive and develop human-like relationships with smart objects grounded in varying social roles.

Traditionally, machines tend to be viewed and developed as servants who deliver a service and aid humans in completing a task. Common examples of the servant role include robots designed to help older people navigate their daily activities (Di Nuovo et al., 2018) and robots that provide a snack delivery service in university buildings (Lee et al., 2009). However, machines are now becoming increasingly capable of providing emotional support, which opens up the possibility of being companions of users (Sundar et al., 2017). For instance, AV1, a small telepresence robot, has been developed to reduce the loneliness of children and young adults with long-term illnesses. In user-IoT interactions, a hub component connected to the IoT system, like a mobile app, becomes the primary contact point where users interact with IoT. Therefore, we predict that the social role assigned to the virtual agent embedded in the IoT mobile app will have a significant impact on the power relationship between IoT and users. In this view, we examine whether variations in the role (servant vs. companion) of the mobile app agent affect users’ perceptions of social power and subsequent persuasive outcomes.

Previous research on human-robot interaction has shown that these two social roles have distinct effects on users’ responses to a social robot. For example, Dautenhahn et al. (2005) found that people preferred to have robots as servants rather than companions (e.g., friends). In another study, Sundar et al. (2017) found that senior citizens perceived companion robots as more socially attractive than servant robots. Given that servant and companion agents are in different hierarchical relationships with users, users may experience varying levels of social power with the IoT mobile app agent equipped with different roles. When interacting with the smart object, people have a strong desire to control the object, and feelings of mastery contribute to increased interactions and usage (Schweitzer et al., 2019). Therefore, if the IoT mobile agent performs the role of a servant rather than a companion, it will satisfy a greater extent of users’ psychological needs of attaining mastery of the agent, thereby affording proxy agency to users rather than compromising user agency. Hence, servant (vs. companion) IoT will foster a higher level of perceived power in users over IoT. We thus hypothesize that:

H1: Participants will perceive greater power over IoT when its mobile app agent is framed as a servant (vs. a companion).

### The Moderator: The Scope of Internet of Things Controlled by the Mobile App

A mobile app for IoT control can be designed to control a single type of device or an entire IoT system encompassing multiple devices. As for a single device, a typical example is that Philips Hue provides a mobile app for monitoring and controlling the light settings. IoT devices can also be connected to each other and create a networked system (e.g., smart home or smart city) encompassing interconnected multiple smart elements (e.g., Patel et al., 2016). For example, Apple HomeKit and Samsung SmartThings provide mobile apps for users to monitor and control the whole smart home system comprising various smart objects (e.g., air conditioner, fridge). In this sense, IoT is usually treated as a holistic system and provides a centralized platform or application enabling users to interact with the whole system.

Derived from the assemblage theory (DeLanda, 2016), Hoffman and Novak (2018) suggested that a collection of smart devices can create an assemblage (e.g., smart home), and smaller assemblages can be nested in larger assemblages. For example, a smart security camera can be bounded by a home security assemblage comprising a smart security camera and a home security mobile app. This security camera-mobile app assemblage can also work as a part of a larger smart home assemblage, being connected to other smart components, including a smart thermostat, TV, speaker, lights, etc. Thus, users can interact with either a part of the assemblage (e.g., mobile app-security camera assemblage) or the whole assemblage (e.g., smart home). Hoffman and Novak (2018) explained that different user experiences could emerge from such different scopes of interactions (i.e., user-part vs. user-whole). Under this view, the size of the assemblage that users get to communicate with through the IoT mobile app may influence user experience with servant vs. companion IoT, specifically through delineating the scope of IoT where the mobile app agent can exert influence on.

According to French and Raven (1959), the scope of one’s controllability is a crucial determinant of power. In the context of IoT, the scope of IoT assemblage controlled by the mobile app agent defines the agent’s capacities to influence how the IoT works. When the user-IoT relationship is defined as a vertical (i.e., master-servant) relationship, the scope of the controllability that the mobile app agent has will be easily translated to the users’ perceived control over IoT. Hence, if the scope of IoT assemblage controlled by the servant agent becomes larger, users will feel greater power over IoT. However, when the user-IoT relationship is situated on a horizontal (i.e., companion) relationship, users will perceive they interact with a more or less equivalent partner. Therefore, if the IoT assemblage controlled by the companion agent becomes larger, users may feel even lesser control over the companion agent and may feel agency conflict with IoT; the larger the IoT assemblage controlled by the companion agent, the smaller social power users would feel. Following this, it is reasonable to infer that the scope of IoT that the mobile app controls (smart fridge vs. smart home) will moderate the effect of social roles attached to the IoT mobile app agent on perceived power over IoT, with the larger scope rendering a greater difference in perceived power between servant and companion roles.

H2: There will be a significant interaction between the scope and social role of IoT on participants’ perceived power over IoT; the difference in participants’ perceived power that servant vs. companion IoT agent elicits will be greater when the app controls a smart home (vs. a fridge).

### Persuasive Effects of Power Dynamics *via* Influencing Perceived Threat-to-Freedom

The power dynamics in the relationship between a message source and receiver have a significant implication on persuasive effects. The reactance theory (Brehm, 1966) explains that persuasive efforts can be regarded as a threat to one’s freedom, thus producing undesired effects (e.g., Dillard and Shen, 2005). When one feels that his freedom is threatened by the persuasive message, self-defense motivation is activated, thereby eliciting negative emotions and cognitions (Brehm, 1966; Dillard and Shen, 2005). As such, perceived threat-to-freedom serves as a significant antecedent to reactance resulting in a less persuasive effect (e.g., Dillard and Shen, 2005; Quick, 2012).

By extension, the level of power users perceive in the relationship with IoT is expected to affect the effectiveness of persuasive messages from IoT. An early study has demonstrated that a message from a high-power source, compared to a low-power source, produced less persuasive effects (Pennebaker and Sanders, 1976). Studies also found that the high-power source reflected in persuasive messages using forceful or controlling language induced negative cognitions and anger, resulting in less desirable attitudes and behaviors among message receivers (e.g., Dillard and Shen, 2005; Quick and Considine, 2008).

Internet of things has a great potential for health promotion and health care. Smart home IoT technologies can be used for overall health monitoring and support systems for various groups of users with different needs (e.g., Rialle et al., 2002; Deen, 2015). Hence, receiving health-related messages or promotions can become a common type of interaction between IoT and users. For example, a smart fridge can recommend healthy recipes and create a shopping list for a healthier diet by tracking its contents and users’ eating habits (Gu and Wang, 2009). Bridging the new social power dynamics in human-object interactions to the basic tenet of the reactance theory, we hypothesize that:

H3. As participants’ perceived power increases, they will be less likely to feel that the health message from IoT is a threat to their freedom, leading to better persuasive effects.

Next, combining H1 and H3, it is hypothesized that:

H4: The servant (vs. companion) IoT will lead to higher persuasive effects *via* increasing perceived power in users and thus reducing perceived threat-to-freedom.

Lastly, combining H2 and H3, we hypothesize the moderated mediation effects:

H5: The scope of IoT (i.e., home vs. fridge) will moderate the indirect effects of the social role of IoT (i.e., servant vs. companion) on persuasive effects mediated by two consecutive mediators—perceived power and threat-to-freedom.

## Methods

### Experimental Design and Apparatus

A 2 × 2 between-subjects online experiment, with four conditions representing the two types of roles (servant vs. companion) and two levels of IoT scope (one IoT device; i.e., smart fridge vs. entire IoT network; i.e., smart home), was conducted. The researchers developed a web-based mobile application featuring a virtual assistant to allow participants to interact with a fictitious smart refrigerator and smart home named Genie-Fridge and Genie-Home, respectively. To have the participants experience the agentic capacity of IoT, we designed the application to recommend recipes, create a grocery shopping list based on the recipes and the content in the fridge, and finally provide a healthy nutrition message with a recommendation of related shopping items. During the experiment, participants used their mobile devices (e.g., smartphones, tablet computers) to interact with the application and complete the experimental task.

### Participants

A total of 347 participants residing in the United States were recruited from Amazon Mechanical Turk. Each participant was compensated with USD 2.50. After screening out those who completed the study too fast (<200 s; *n* = 6), univariate outliers on perceived power (*n* = 2), and those who responded that they did not use a mobile device for study participation (*n* = 5), we used data from 334 respondents (168 males and 166 females; aged 18–73, *M* = 37.8, *SD* = 11.32) for the analysis. The majority of the participants were Caucasians (*n* = 262; 78.4%), followed by Asians (*n* = 37; 11.1%), African Americans (*n* = 29; 78.4%), and other races (*n* = 6; 1.8%). A total of 152 (45.5%) participants responded that they own smart IoT devices at home. Among them, 120 participants owned smart speakers, 76 owned smart lights, 61 owned smart security devices (e.g., security camera, smart lock), 50 owned smart plugs, 49 owned smart thermostats, 39 owned smart remote controllers, and 8 owned smart refrigerators. Participants were randomly assigned to one of the four conditions, and gender distribution in each condition was quite even. See Table 1 for the number of participants and gender distribution in each condition.

**TABLE 1:** Number of participants and gender distribution in each condition.

	Smart home	Smart fridge	Total
Servant	82 (M: 39; F: 43)	90 (M: 41; F: 49)	172 (M: 80; F: 92)
Companion	82 (M: 40; F: 42)	80 (M: 48; F: 32)	162 (M: 88; F: 74)
Total	164 (M: 79; F: 85)	170 (M: 89; F: 81)	334 (M: 168; F: 166)

### Manipulation of Internet of Things Social Role

In the servant condition, participants interacted with a virtual agent that resembled the appearance of a butler. The agent conveyed messages to participants by using deferential language, calling the participant “master” (e.g., “Please kindly review the grocery shopping list.”). In contrast, participants in the companion condition interacted with a virtual agent with more a casual appearance using informal language (e.g., “Review the grocery shopping list.”), calling the participant “friend” or “buddy.”

### Manipulation of Internet of Things Scope

**FIGURE 1:**
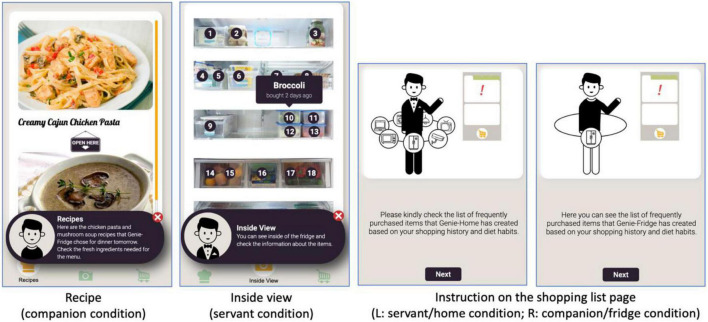
Screenshots of IoT mobile application.

Participants in the one IoT device (smart fridge) condition interacted with Genie-Fridge, a smart refrigerator capable of monitoring items in the fridge, generating recipes, and placing grocery orders using the mobile app. In the entire IoT network condition (smart home), participants were introduced to Genie-Home and its basic functions, such as monitoring and controlling various features of and appliances, including the refrigerator, in the smart home. They were told that they could use Genie-Home to scan items in the fridge, generate recipes, and place grocery orders, just like what has been explained in the smart fridge condition. See Figure 1 for the screenshots of the mobile app.

### Procedure

**FIGURE 2:**
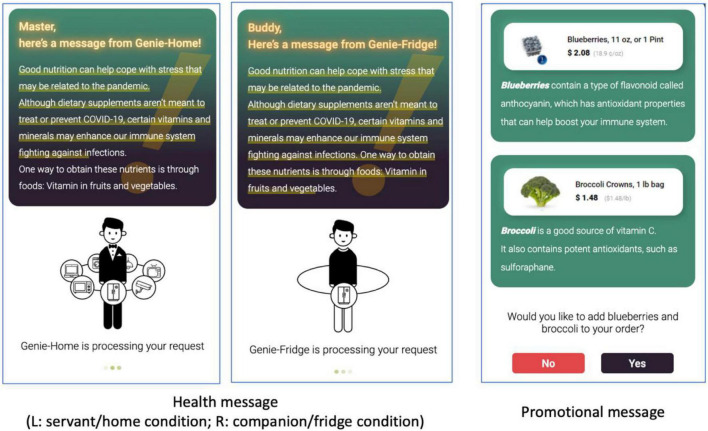
Persuasive components of IoT mobile application.

Participants were informed that they would interact with a mobile application for a smart refrigerator/home and report their overall experiences and evaluations of a smart refrigerator/home. They were also asked to use a mobile device (i.e., smartphone or tablet computer) for participation. After signing an informed consent form, participants were asked which device they were currently using. Those who did not choose either smartphone or tablet computer were screened out. Then, each participant was randomly assigned to one of the four experimental conditions. In the main experiment, Genie-Fridge/Home first greeted participants, briefly explained its features, and provided recipes for chicken pasta and mushroom soup (i.e., the menu it has selected for the next day’s dinner). It then created a shopping list based on the ingredients needed to prepare the dinner and the items already in the fridge and asked participants to confirm the list and place an order. While processing the purchase request, participants were presented with a short message about how vitamins and minerals obtained from fruits and vegetables could enhance the immune system fighting against COVID-19, adapted from the Centers for Disease Control and Prevention [CDC] (2020) (Figure 2, left). After reading the message, participants were asked to choose whether they wished to add blueberries and broccolis beneficial for the immune system to the grocery order they had placed by clicking the “yes” or “no” button (Figure 2, right). Then, they were directed to the survey questionnaire.

### Measures

Participants were asked to indicate the *level of power* that the participant and Genie Fridge (Genie Home) have in the interactions between the participant and the smart home/fridge on a slider scale ranging from −100 to 100. −100 was labeled as “Genie [Home/Fridge] has all the power,” and 100 was labeled as “I have all the power” [*M* = 58.91, *SD* = 41.48, Range: −79 to 100; adopted from Wolfe and McGinn (2005)].

*Threat-to-freedom* that participants felt by the message was assessed with four items (e.g., “The health message threatened my freedom to choose”; Dillard and Shen, 2005) using 7-point Likert scales ranging from 1: strongly disagree to 7: strongly agree (α = 0.88; *M* = 2.47; *SD* = 1.35).

*Persuasive effects* were assessed with three outcomes: attitude, behavior intention, and actual behavior. With five items, participants were asked about their *attitude* toward consuming fruits and vegetables (e.g., blueberries and broccoli) to boost their immune system (e.g., harmful-beneficial, undesirable-desirable) using a 7-point bipolar scale (α = 0.89; *M* = 6.27; *SD* = 0.92). On two 7-point Likert scale items (1: strongly disagree to 7: strongly agree), participants rated their *intention* to consume blueberries and broccoli often to boost their immune system (*r* = 0.66; *M* = 5.14; *SD* = 1.43). For the *behavior* measure, the log data extracted from the application were used in the analysis. 77.5% (*n* = 259; coded as “1”) added blueberries and broccoli to the order list, while 22.5% (*n* = 75; coded as “0”) did not add them to the list.

## Results

### Manipulation Check

Two-way analyses of variance were conducted to examine whether the experimental manipulations were successful. To test the IoT social role (servant vs. companion) manipulation, participants were asked to rate the following item using a 7-point bipolar scale: “The character on the application was more like my [servant (1)…friend (7)].” Participants in the companion condition (*M* = 3.81, *SD* = 1.80) rated significantly higher on this item than those in the servant condition (*M* = 3.12, *SD* = 1.82), *F*_(1,330)_ = 12.10, *p* < 0.001, η^2^ = 0.04. To verify the manipulation for the IoT scope (home vs. fridge) employed in the study, participants were asked to rate the item stating, “The application I interacted with was designed to control [a smart device (1)…a smart home having multiple smart devices (7)],” on a 7-point bipolar scale. As expected, the smart home condition (*M* = 4.66, *SD* = 2.29) displayed a significant higher rating on this item than the smart fridge condition (*M* = 2.68, *SD* = 2.06), *F*_(1,330)_ = 71.25, *p* < 0.001, η^2^ = 0.18. There was no significant interaction between the two manipulations.

### Hypothesis Testing

**FIGURE 3:**
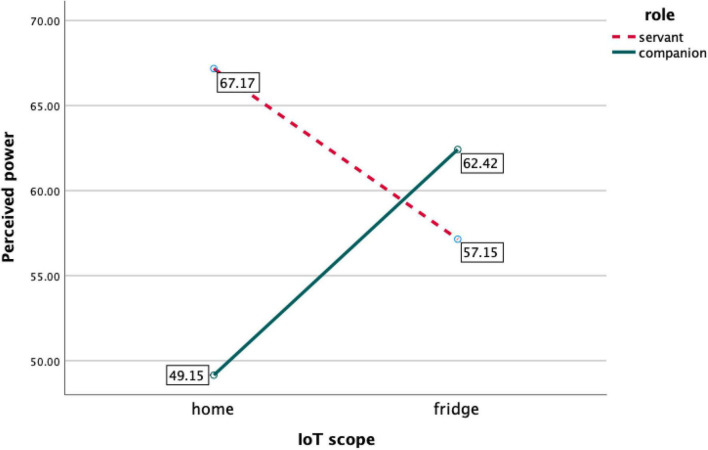
Interaction between social roles and scope of IoT on perceived power.

A two-way analysis of covariance was employed to test the first two hypotheses predicting the main effect of IoT social role (H1) and the interaction between the social role and the scope of IoT on perceived power (H2). Gender and age were entered as covariates. Studies have shown that age is the significant factor that moderates the perceptions and adoption of new technology (e.g., Morris and Venkatesh, 2000; Barnard et al., 2013). Similarly, gender is known to influence how people use and perceive new technology (e.g., Venkatesh et al., 2000; Li et al., 2008). In addition, since we used a male character for the visual representation of the virtual assistant, participants’ gender was controlled in the analysis, as they may respond differently to the male assistant in accordance with their gender identity. The results indicated that there was no significant difference between servant (*M* = 62.13, *SD* = 41.30) and companion (*M* = 55.49, *SD* = 41.52) conditions in terms of perceived power, *F*_(1,328)_ = 1.99, *p* = 0.16, η^2^ = 0.01. Thus, H1 and H4 (which was based on H1) were not supported. Similarly, the smart home (*M* = 58.10, *SD* = 42.48) and fridge (*M* = 59.69, *SD* = 40.6) conditions did not produce a significant difference in perceived power, *F*_(1,328)_ = 0.13, *p* = 0.72, η^2^ = 0.00. However, there was a significant interaction between the social role and the scope of IoT on perceived power, *F*_(1,328)_ = 6.61, *p* = 0.01, η^2^ = 0.20. The covariates did not have significant effects on perceived power (gender: *F*_(1,328)_ = 0.028, *p* = 0.87; age: *F*_(1,328)_ = 2.48, *p* = 0.12). A *post hoc* test employing Bonferroni adjustments showed that when the participants interacted with a mobile app agent for a smart home, they perceived a significantly higher level of power when it was framed as a servant (*M* = 67.17, *SE* = 4.55) than a companion (*M* = 49.15, *SE* = 4.54; *p* = 0.005). However, when they interacted with a mobile app for a smart fridge, the difference was not statistically significant (servant: *M* = 57.15, *SE* = 4.35; companion: *M* = 62.42, *SE* = 4.62; *p* = 0.41). Therefore, H2 was supported. Figure 3 presents the interaction pattern.

Model 4 of PROCESS macro (Hayes, 2018) was used to test if the perceived power of the participants enhances persuasive effects *via* suppressing perceived threat-to-freedom. PROCESS estimates values using ordinary least squares (OLS) regression for continuous dependent variables (i.e., attitude and intention in this study) and logistic regression for the dichotomous outcome (i.e., behavior in this study). The results indicated that the indirect effect of perceive power on attitude (*B* = 0.35, *SE* = 0.14, 95% CI 0.12–0.65) intention (*B* = 0.55, *SE* = 0.22, 95% CI 0.19–1.03) and behavior (*B* = 0.48, *SE* = 0.25, 95% CI 0.09–1.06) were all significant *via* perceived threat-to-freedom, supporting H3. No significant direct effect was found.

**FIGURE 4:**
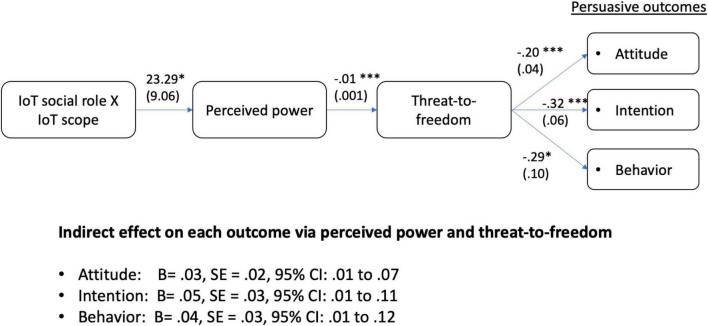
Interaction between IoT social role and scope on persuasive outcomes *via* perceived power and threat-to-freedom. Unstandardized coefficient B (Standard Error); *p < 0.05, ***p < 0.001; gender, age, and main effects of IoT social role and scope were entered as covariates.

We then tested if perceived power and perceived threat-to-freedom mediated consecutively in the interaction between the social role and the scope of IoT on persuasive outcomes, using Model 6 of PROCESS macro (Hayes, 2018). The interaction term was entered as the independent variable; perceived power and threat-to-freedom were entered as serial mediators. The results revealed that the interaction between social role and the scope of IoT had significant influences on attitude, intention, and behaviors, mediated by the two consecutive mediators. Hence, H5 was supported. Figure 4 summarizes the results.

## Discussion

Our study contributes to the human-IoT interaction literature by demonstrating how a source factor (i.e., social role) and a technological factor (i.e., control scope) of an IoT agent influence its persuasive effects on users. The study also validates perceived power and threat-to-freedom as the psychological mechanisms underlying the persuasive effects of IoT.

This study extends the significant role of source factors in persuasion (e.g., Chaiken, 1980) into the context of human-IoT communication. In line with the CASA paradigm (Reeves and Nass, 1996), our results showed that users reacted to the persuasive message from IoT differently in accordance with the social role (i.e., servant vs. companion) assigned to the embodied mobile app agent. When interacting with a smart home *via* the mobile app, participants felt a higher level of power over the servant, compared to the companion, IoT as they would when interacting with a human servant or companion, which led to less threat-to-freedom and thus greater persuasive effects. However, we identified a boundary condition of this effect. The difference between servant and companion IoT agent on perceived power over IoT held only when the embodied agent was designed for controlling a large scope of IoT (i.e., smart home in our study); when the agent is for controlling only one device (i.e., smart fridge), the level of perceived power over IoT did not vary as a function of the social role assigned to the IoT agent. By being embedded in a device connected to the IoT network, a virtual agent functions as the most visible and proximate source among the multiple technological elements in the IoT network. The mobile IoT agent also allows users to interact with their IoT network anytime and anywhere, making source cues attached to the mobile app agent have a prominent impact on the communication outcomes of the IoT. However, our study result indicates that the technological capacity of IoT also plays an integral role in defining the social power of and social relations with IoT by modulating the effect of the source (agent) factor.

By linking the psychological tension arising from interacting with autonomous technology (e.g., Jia et al., 2012, 2013; Sundar, 2020) and the reactance theory (Brehm, 1966), our study identified the tension between machine vs. human agency as the psychological mechanism explaining how IoT can influence its users’ perceptions and behaviors. Human-computer interaction (HCI) literature (e.g., Jia et al., 2012, 2013; Sundar, 2020) suggests that when humans interact with an advanced technology capable of exercising own agency, they may feel that their agentic capacity is compromised by the machine, thus experiencing psychological tension (e.g., threat-to-freedom). In line with the reactance theory, participants exhibited greater reactance toward the persuasive message when they felt that their agentic power was interrupted by IoT. More importantly, our findings extend the reactance theory by revealing that the interplay between source features and technological capacity can influence people’s psychological reactance to message advocacy. While most previous research has examined the impact of message features on the perceived threat-to-freedom (Quick et al., 2012), this study demonstrates that the scope of the IoT system can influence how users react to persuasive messages conveyed by IoT serving as an information source. In sum, this study contributes to communication and HCI research by showing how IoT can function as a communication source in various types of strategic communication.

This study also provides valuable practical implications for communication practitioners aiming to incorporate IoT technology into their practices. We found that the servant role produces more positive persuasive effects in the smart home context. This result suggests that, for greater persuasive effects, the virtual smart home agent should be framed as a subordinate (e.g., servant, assistant, or helper) with a more formal and respectful tone of communication, thereby providing users with greater perceptions of social power. Moreover, the psychological tension between machine and human agency can be alleviated when users are empowered with great power in the interaction. IoT developers can design a thoroughly user-centric interface and provide users with more power to control connected devices.

Limitations of this study should be noted. Participants only engaged in a brief, one-time interaction with the mobile IoT agent, suggesting that users perceive and respond differently to IoT when involved in long-term usage of IoT. As Hoffman and Novak (2018) suggest, users’ experience and relationships with IoT may evolve as they interact with it over time. By employing a longitudinal approach, future research may explore how the social power dynamics between users and IoT evolve long-term and investigate its implication in persuasive communication.

In addition, our study tested the hypotheses only with a male agent, but participants may have shown different responses to the gender of the agent, especially in accordance with the match (or mismatch) between the character and the participant’s gender. By entering participants’ gender in the analysis as a covariate, we have controlled for the gender congruency effects. However, future research can test whether the gender cues influence the persuasiveness of IoT *via* perceived power for more nuanced design implications. In addition, unlike a servant, a companion may imply an affective relationship with the user rather than a power relationship. However, given that the participants did not have a chance to build relationships with the virtual agent prior to our experiment, we assumed that they did not build an affective attachment with the agent. Hence, affective relationships that participants may have experienced with the agent would not have influenced the results significantly. However, in future studies, the cultural and relational meanings and values attached to social roles of IoT, such as servants and companions, should be more fine-grained.

## Conclusion

This study demonstrated how the power relationship between smart home IoT and users can be formulated and how the power relations shape the persuasiveness of IoT as a communication source. The results showed that the human-IoT power relationship varied as a function of the social roles attached to the IoT agent and the scope it controls. Supporting the CASA paradigm, we found that the power relationships that people perceive from others having different social roles and different levels of control can be transferred to the human-IoT interactions. Moreover, the study also validated perceived power and threat-to-freedom as the psychological mechanisms underlying the persuasive effects of IoT. For a better utilization of smart IoT as a persuasive agent, it is imperative to consider the power relationships developed between human users and smart objects.

## Data Availability Statement

The raw data supporting the conclusions of this article will be made available by the authors, without undue reservation.

## Ethics Statement

The studies involving human participants were reviewed and approved by Nanyang Technological University. The patients/participants provided their written informed consent to participate in this study.

## Author Contributions

HK and KK contributed to the conception and design of the study. HK performed the statistical analysis. All authors wrote the first draft and sections of the manuscript and contributed to the manuscript revision, read, and approved the submitted version.

## Conflict of Interest

The authors declare that the research was conducted in the absence of any commercial or financial relationships that could be construed as a potential conflict of interest.

## Publisher’s Note

All claims expressed in this article are solely those of the authors and do not necessarily represent those of their affiliated organizations, or those of the publisher, the editors and the reviewers. Any product that may be evaluated in this article, or claim that may be made by its manufacturer, is not guaranteed or endorsed by the publisher.
